# Low lumbar multifidus muscle status and bone mineral density are important risk factors for adjacent segment disease after lumbar fusion: a case–control study

**DOI:** 10.1186/s13018-022-03388-8

**Published:** 2022-11-16

**Authors:** Zhaoyang Gong, Dachuan Li, Fei Zou, Siyang Liu, Hongli Wang, Xiaosheng Ma

**Affiliations:** Department of Orthopedics, Huashan Hospital, Fudan University, No. 12 Urumqi Middle Road, Jing’an District, Shanghai, China

**Keywords:** Multifidus muscle, Bone mineral density, Adjacent segment disease

## Abstract

**Background:**

The quantity and quality of the paraspinal muscles are important factors that lead to spinal diseases. However, the role of paraspinal muscles in the pathogenesis of adjacent segment disease (ASD) after lumbar fusion surgery is rarely studied. The purpose of the research is to investigate the relationship between paraspinal muscles and ASD.

**Methods:**

Thirty-three patients with ASD were included, and 33 controls without ASD were matched according to the basic demographic information. Cross-sectional images of the paraspinal muscles at each intervertebral disk level (L1–S1) before the first operation were analyzed, and the cross-sectional area (CSA) and degree of fat infiltration (FI) of the multifidus (MF) muscle and the erector spinae muscle were compared.

**Results:**

There was no significant difference in demographic characteristics (*P* > 0.05) except for the bone mineral density (BMD) (*P* = 0.037) between the two groups. There were significant differences in the CSA and FI of the lower lumbar multifidus (*P* < 0.05). The CSA of the MF muscle at L3–L4, FI of the MF muscle at L4–L5 and L5–S1 and BMD were important risk factors for ASD. Among patients who received two-segment fusion for the first time, significant difference was observed in the degree of FI of the MF muscle in the lower lumbar segment (*P* < 0.05).

**Conclusions:**

The CSA, FI and BMD of the lower lumbar MF muscle were closely related to the occurrence of ASD. The CSA of the MF muscle at L3–L4, the degree of FI of the MF muscle at L4–L5 and L5–S1 and BMD were important risk factors for ASD. The number of fusion segments in the first operation has a certain impact on the above-mentioned conclusions.

## Background

ASD after lumbar fusion is diagnosed by the imaging of the adjacent fusion segments and the presentation of corresponding clinical symptoms. With the extensive development of lumbar fusion surgery, scholars have conducted a series of studies on the risk factors of ASD. At present, the overall consensus is that age, sex, body mass index (BMI), spinal-pelvic balance parameters, the number of fusion segments in the first operation and surgical approach are closely related to the occurrence of ASD [1–4].

In recent years, people have gradually found that the quantity and quality of the paraspinal muscles are important factors that lead to spinal diseases. At present, a large number of studies have defined the role of paraspinal muscle in low back pain, neurological function, proximal junctional kyphosis and physical function [5–9]. The method for evaluating paraspinal muscles is to measure the CSA or FI of the selected cross section of the muscles with the help of patients’ MRI images to indirectly reflect the quantity and quality of the muscles.

However, there are few studies on the relationship between paraspinal muscles and ASD. Chang [10] et al. and Kim [11] et al. demonstrated that preoperative smaller CSA of the paraspinal muscle is a risk factor for postoperative ASD. However, in both studies, only a single-level cross section (L4–L5) was selected for muscle measurement. On the other hand, Duan [12] et al. used the Goutallier method to visually measure the paraspinal muscles’ degree of FI and proved its connection with ASD. As the FI was not quantitatively evaluated, the accuracy may be low.

On the basis of previous studies, this study adopted the principle of 1:1 matching to conduct a case–control study. With the help of professional image analysis software, the CSA and FI of the paraspinal muscles at all lumbar intervertebral disk levels were quantitatively measured to explore whether the size and quality of the paraspinal muscles are risk factors for ASD after fusion.

## Methods

### Subject population

The ethics review committee of Huashan Hospital affiliated with Fudan University approved this single-center retrospective study. As the identity of the patients was anonymous, the requirement for an informed consent form was waived.

From May 2006 to September 2021, we included 67 patients who underwent posterior lumbar decompression, fusion and internal fixation because of ASD in our hospital; both operations were performed by the same surgical team in our hospital. The diagnostic criteria of ASD were as follows: Imaging showed that the slippage of the vertebral body on lateral film was ≥ 4 mm, the range of motion of the adjacent segments was more than 10°, and the loss of intervertebral disk height was more than 10% [13, 14]; MRI indicated that the modified Pfirrmann grade of the intervertebral disk [15] was grade IV or V, or there was obvious intervertebral disk herniation and lumbar spinal canal stenosis at the adjacent segments; the corresponding clinical symptoms manifested based on the above-mentioned imaging findings. The surgical indications of ASD were low back pain, nerve root symptoms and intermittent claudication, which seriously affected the quality of life, and there was no improvement after three months of conservative treatment. Exclusion criteria were: 1. lack of complete and clear MRI images before operation; 2. the follow-up period was less than 2 years; and 3. the number of fusion segments in the first operation was 3 or more. In the final case group, 34 cases were excluded, and 33 patients were included. Using a 1:1 matching method, 33 controls were matched according to sex, age at the time of operation, operation segment and follow-up time, selected from May 2006 to September 2021, patients who underwent posterior lumbar decompression, fusion and internal fixation in our department. The inclusion criteria of the control group were as follows: 1. Posterior lumbar decompression, fusion and internal fixation were performed in our hospital because of lumbar degenerative diseases; 2. no secondary operation was performed on the lumbar vertebrae. The exclusion criteria were as follows: 1. lack of complete and clear preoperative MRI images; 2. during the postoperative follow-up, the fusion adjacent segments showed obvious degeneration; and 3. the patients’ clinical symptoms were significantly worse than those experienced after the previous operation.

### Lumbar MRI scan acquisition and images analysis

The preoperative MRI images of the lumbar spine were obtained by the following machines in the supine position: a 3 T MRI scanner (Siemens Magnetom Verio, Erlangen, Germany). The imaging parameters were as follows: repetition time/echo time: 3500/118 ms; slice thickness: 4 mm; intersection gap: 0.4 mm; matrix: 336 × 384; and field of view: 250 mm.

The T2-weighted images of the median cross section of each intervertebral disk in the patient’s L1–S1 were derived from a PACS workstation (Centricity Radiology RA100, GE Healthcare) in JPEG format, and the image information was deeply mined by image processing software (ImageJ, version 2, National Institutes of Health).

**Fig. 1:**
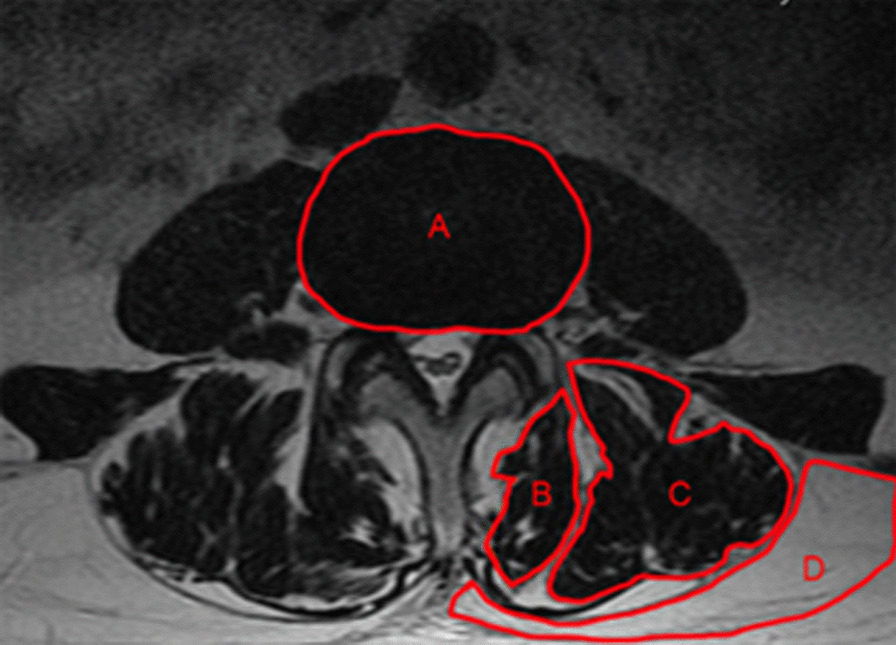
The outline of the intervertebral disk (**A**), multifidus muscle (**B**), erector spinae muscle (**C**) and subcutaneous fat (**D**) are represented by hand-drawn region of interest using Image J

Referring to the previous method proposed by Hyun [16] et al., the area and gray value of the paraspinal muscles (MF muscle and ES muscle) in each patient were measured, and the average values were measured on the left and right sides. Using the ImageJ hand-drawn region of interest (ROI) function, we selected the MF muscle, ES muscle, intervertebral disk and subcutaneous fat on these five levels. The CSA of the paraspinal muscle/intervertebral disk CSA*100 was used as the relative CSA (rCSA) of the paraspinal muscle to eliminate the difference in body weight among different individuals, and the gray value of the paraspinal muscle/subcutaneous fat * 100 was used as the relative FI (rFI) to eliminate individual differences and quantitatively analyze the degree of fat infiltration (Fig. 1).

In addition to this, we assessed the degree of disk degeneration in the adjacent segments of the fused segment with the help of the Pfirrmann classification on MRI sagittal images [15].

### X-ray and CT images analysis

**Fig. 2:**
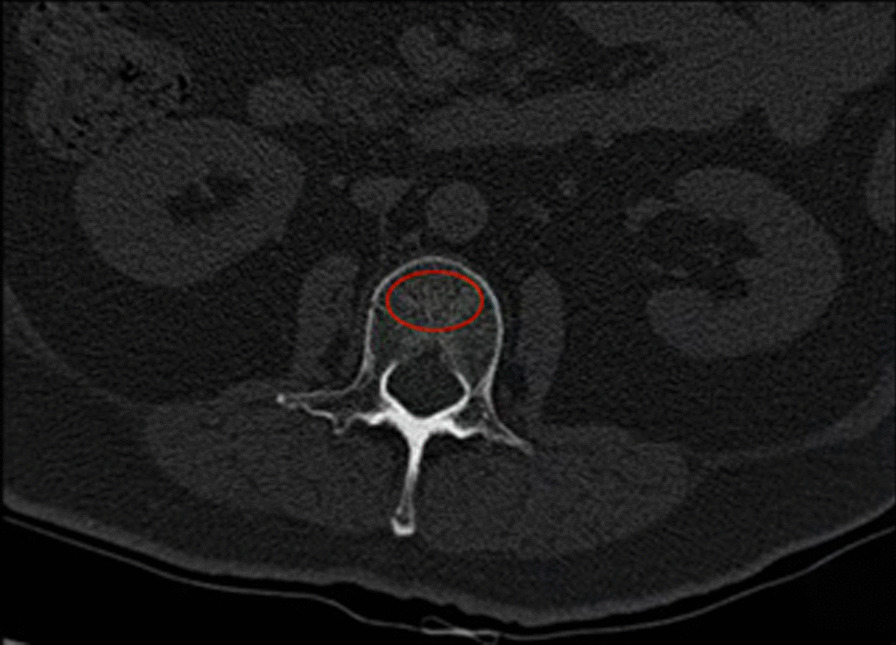
Place an as-large-as-possible oval ROI in the cancellous tissue of the vertebral body, and read out the average CT value of this area directly to indicate the bone mineral density of the patient

We found the patient’s preoperative X-rays and CT images in the PACS system and used its own tool to measure the following parameters. For X-ray images, we measured the lumbar lordosis (LL) and sacral slope (SS) to represent the sagittal balance of the lumbar spine. LL angle was defined as the Cobb angle formed between the superior edge of L1 and the superior edge of the sacrum; SS was defined as the angle between the upper edge of the sacrum and the horizontal line. For CT images, CT values were used to express BMD. The median cross-sectional images of the L1 and L2 vertebrae were selected, and an as-large-as-possible oval ROI was placed in front of the cancellous bone of the vertebral body. The average CT value of this area was read directly, and then, the patient’s bone mineral density was represented by the average value of the L1 and L2 measured data (Fig. 2).

To reduce measurement bias, each patient’s images were measured by two experienced spinal surgeons and the measurement results were averaged.

### Statistical analysis

Data processing software (SPSS, version 23.0 for Windows, IBM) was used to analyze the data. For demographic data, the patient’s age at the first operation, the time of follow-up, the preoperative LL angle and SS and the CT value of vertebral cancellous bone before the first operation between ASD patients and controls were compared by paired sample t test. The chi-squared test was used to compare the sex differences and specific surgical segments between the two groups. The rank sum test was used to compare the number of initial fusion segments and preoperative adjacent segment disk Pfirrmann’s grade between the two groups. Paired sample t test was used to compare the rCSA and the rFI of each segment of the multifidus and erector spinae muscle of the two groups before the first operation, and a univariate regression analysis was used to analyze the risk factors. *P* < 0.05 was defined as a statistically significant difference.

## Results

As shown in Table 1, the average age of the patient at the first operation in the 33 patients who underwent the second operation for ASD was 62.31 ± 10.80 years, while that of the control group was 62.9 ± 10.73 years. There was no significant difference between the two groups (*P* > 0.05). In addition, there was no significant difference in the ratio of males to females, the specific segment of the first fusion operation, the preoperative LL angle, the preoperative SS, the preoperative Pfirrmann’s grade at the adjacent segment of surgery and the follow-up time between the two groups (*P* > 0.05). However, the CT value of vertebral cancellous bone in patients with ASD was significantly lower than that in the control group (*P* = 0.037).

**Table 1:** Demographic characteristics

Characteristic	Patients with ASD (*n* = 33)	Control (*n* = 33)	*P* value
Age at surgery, (yrs), mean ± SD	62.30 ± 10.80	62.90 ± 10.73	0.820
Gender, *n* (%)			1.000
Male	14 (42.4)	14 (42.4)	
Female	19 (57.6)	19 (57.6)	
Bone density, HU value, mean ± SD	113.55 ± 36.66	133.13 ± 38.11	0.037*
Preoperative LL, (°), mean ± SD	52.40 ± 7.17	50.24 ± 6.98	0.218
Preoperative SS, (°), mean ± SD	34.88 ± 5.21	35.26 ± 5.84	0.778
Number of fusion segments, *n* (%)			1.000
1	16 (48.5)	16 (48.5)	
2	17 (51.5)	17 (51.5)	
Preoperative Pfirrmann’s grade at the proximal adjacent segment of surgery, n (%) 0.769			
I	1	1	
II	5	3	
III	15	19	
IV	8	9	
V	4	1	
Preoperative Pfirrmann’s grade at the distal adjacent segment of surgery, *n* (%) 0.304			
I	0	0	
II	1	1	
III	8	11	
IV	6	3	
V	0	0	
Initial fusion segment, *n* (%)			1.000
L3–4	5 (15.2)	5 (15.2)	
L4–5	3 (9.1)	3 (9.1)	
L5–S1	8 (24.2)	8 (24.2)	
L3–5	7 (21.2)	7 (21.2)	
L4–S1	10 (30.3)	10 (30.3)	
Follow-up years, (yrs), median (IQR)	5.2 (4.0)	5.0 (4.1)	0.639

Mean values are given as mean ± SD

^*^Statistical significance

ASD indicates adjacent segment disease; LL, lumbar lordosis; and SS, sacral slope

A *P* value of < 0.05 was considered to indicate statistical significance

Table 2 shows the rCSA of the paraspinal muscle at the median cross-sectional level of each intervertebral disk in the ASD group and the control group before the first operation. It can be seen from the table that there was no significant difference in the rCSA of the MF muscle between the two groups at L1–L2 and L2–L3. At L3–L4, L4–L5 and L5–S1, the rCSA of the MF muscle in the ASD group was lower than that in the control group, and the difference was statistically significant (*P* < 0.05). For the ES muscle, there was no significant difference in rCSA between the two groups at each segmental level.

**Table 2:** Relative cross-sectional area (rCSA) for multifidus (MF) muscle and erector spinae (ES) muscle

Level	Muscle	Patients with ASD (*n* = 33)	Control (*n* = 33)	*P* value
L1–2	MF	22.79 ± 8.77	21.50 ± 7.65	0.528
	ES	117.05 ± 18.92	110.95 ± 18.01	0.185
L2–3	MF	28.79 ± 10.88	29.85 ± 8.69	0.663
	ES	108.06 ± 12.91	105.77 ± 10.65	0.435
L3–4	MF	28.42 ± 7.23	32.52 ± 7.76	0.030*
	ES	86.66 ± 19.00	85.23 ± 22.18	0.780
L4–5	MF	29.85 ± 8.18	34.54 ± 6.60	0.013*
	ES	71.38 ± 13.77	71.58 ± 16.65	0.958
L5–S1	MF	29.26 ± 7.59	34.21 ± 9.41	0.022*
	ES	51.18 ± 20.73	51.14 ± 20.70	0.993

The values are given as mean ± SD

^*^Statistical significance

ASD indicates adjacent segment disease; rCSA, relative cross-sectional area; ES, erector spinae; and MF, multifidus

A *P* value of < 0.05 was considered to indicate statistical significance

As shown in Table 3, at L3–L4, L4–L5 and L5–S1 levels, the rFI of the MF muscle in the ASD group was significantly higher than that in the control group (*P* < 0.05). There was no significant difference in the rFI of the ES muscle between the two groups (*P* > 0.05).

**Table 3:** Relative fat infiltration (rFI) for multifidus (MF) muscle and erector spinae (ES) muscle

Level	Muscle	Patients with ASD (*n* = 33)	Control (*n* = 33)	*P* value
L1–2	MF	35.05 ± 8.94	31.72 ± 6.07	0.082
	ES	33.03 ± 8.12	30.98 ± 7.27	0.284
L2–3	MF	35.35 ± 6.14	32.64 ± 5.83	0.071
	ES	31.86 ± 6.36	30.56 ± 6.82	0.426
L3–4	MF	38.97 ± 6.01	32.12 ± 6.89	< 0.001*
	ES	34.83 ± 7.68	31.60 ± 6.84	0.076
L4–5	MF	41.58 ± 6.26	32.07 ± 7.13	< 0.001*
	ES	36.59 ± 8.55	32.78 ± 7.77	0.062
L5–S1	MF	38.75 ± 7.61	32.93 ± 6.51	0.001*
	ES	38.43 ± 6.73	36.56 ± 8.38	0.720

The values are given as mean ± SD

^*^Statistical significance

*ASD* indicates adjacent segment disease, *rFI* relative fat infiltration, *ES* erector spinae, *MF* multifidus

A *P* value of < 0.05 was considered to indicate statistical significance

Then, the average values of BMD, rCSA and rFI of the MF at each level were calculated. If the patient’s value is greater than the average, it is defined as a “high level,” and vice versa as a “low level.” Through univariate regression analysis, it was found that rCSA of the MF muscle at L3–L4 (*P* = 0.022, OR = 0.166), rFI at L4-L5 (*P* = 0.005, OR = 17.974), rFI at L5-S1 (*P* = 0.024, OR = 7.140) and BMD (*P* = 0.013, OR = 0.081) were all risk factors for ASD (Table 4).

**Table 4:** Risk factors of the occurrence of ASD

Risk factor	Logistic Regression		
	*P* Value	Odds ratio	95% Confidence interval
rCSA of MF at L3–4 level	0.022*	0.116	0.019–0.729
rFI of MF at L3–4 level	0.337	0.406	0.064–2.562
rCSA of MF at L4–5 level	0.427	0.475	0.076–2.985
rFI of MF at L4–5 level	0.005*	17.974	2.349–137.505
rCSA of MF at L5–S1 level	0.123	0.251	0.043–1.457
rFI of MF at L5–S1 level	0.024*	7.140	1.298–39.292
Bone density	0.013*	0.081	0.011–0.590

^*^Statistical significance

*rCSA* indicates relative cross-sectional area, *rFI* indicates relative fat infiltration, *ES* erector spinae, *MF* multifidus

A *P* value of < 0.05 was considered to indicate statistical significance

Interestingly, when comparing the MF muscle parameters of the two groups according to the segment of the first operation, we found that in the patients undergoing single segment surgery for the first time, the rCSA and rFI of the lower lumbar MF muscle in the ASD group were significantly different from those in the control group, while in the patients undergoing surgery in 2 segments for the first time, there was only a significant difference in the rFI of the MF muscle at L3–L4 and L4–L5 between the two groups (Table 5).

**Table 5:** Comparison of MF characteristics in different levels between the ASD group and the control group when the initial operation segments were 1 and 2, respectively

Level	Characteristic	Number of fusion segments = 1			Number of fusion segments = 2		
		Patients with ASD	Control	*P* value	Patients with ASD	Control	*P* value
L1–2	rCSA	21.50 ± 11.61	20.91 ± 8.51	0.874	23.65 ± 5.85	22.05 ± 7.47	0.506
	rFI	34.38 ± 9.26	31.46 ± 6.84	0.335	35.40 ± 9.41	31.62 ± 5.70	0.179
L2–3	rCSA	25.48 ± 12.54	26.20 ± 6.87	0.847	31.12 ± 8.98	32.64 ± 9.53	0.647
	rFI	34.20 ± 7.43	30.94 ± 5.88	0.193	35.92 ± 5.02	34.16 ± 5.78	0.364
L3–4	rCSA	25.09 ± 6.21	32.52 ± 9.39	0.016*	30.41 ± 7.00	32.17 ± 6.71	0.474
	rFI	37.49 ± 7.02	29.12 ± 6.89	0.003*	40.22 ± 5.19	35.82 ± 4.81	0.019*
L4–5	rCSA	26.23 ± 8.41	34.54 ± 6.92	0.006*	33.43 ± 6.91	34.81 ± 6.79	0.571
	rFI	39.23 ± 6.29	30.51 ± 7.14	0.001*	43.77 ± 5.97	33.99 ± 6.40	< 0.001*
L5–S1	rCSA	29.32 ± 8.29	37.82 ± 8.83	0.011*	29.61 ± 7.58	31.34 ± 8.84	0.556
	rFI	38.43 ± 5.67	31.20 ± 5.11	0.001*	39.45 ± 9.50	34.01 ± 7.49	0.082

The values are given as mean ± SD

^*^Statistical significance

*rCSA* indicates relative cross-sectional area, *rFI* indicates relative fat infiltration, *ES* erector spinae, *MF* multifidus

A *P* value of < 0.05 was considered to indicate statistical significance

## Discussion

With the increasing development and popularity of fusion surgery, the incidence of clinically diagnosed ASD is common. Some studies have shown that the incidence of ASD is between 4 and 31% [17, 18]. Therefore, scholars have performed in depth research on the risk factors for ASD with intentions to prevent its development, improve the patient’s quality of life after surgery and avoid the pain of secondary operations.

In recent years, an increasing number of studies have focused on the relationship between paraspinal muscles and spinal diseases. This is reasonable because the paraspinal muscle itself is connected with the bony structure, can support the body to complete many activities and is a very important anatomical structure of surgical approach, and therefore, details cannot be ignored. At present, there is a lack of a unified method for evaluating the quality and quantity of the paraspinal muscles. It is common to use CSA to indirectly reflect the number of the paraspinal muscles, while FI or functional CSA is used to reflect the quality of paraspinal muscles [19, 20]. Previous studies have proven the relationship between the paraspinal muscle and low back pain, neurological function, proximal junctional kyphosis, physical activity, etc. However, due to the disunity of different measurement methods, research results obtained from different centers sometimes greatly differ and even draw contradictory conclusions. Recently, some scholars began to study the relationship between paraspinal muscles and ASD [10–12]. However, the study has obvious limitations: It may be inappropriate to select only the cross section of the paraspinal muscles at the single segment level to represent the overall paraspinal muscle level because there is no known basic research [21–24] or theoretical support, and there may be poor comparability between different individuals at the same level due to factors such as development, living habits and pathological changes. The accuracy of the FI measurement may be insufficient due to mainly relying on hand-drawn ROI or visual measurement. Based on this, this study measured the cross-sectional parameters of the paraspinal muscles at each level of the lumbar intervertebral disk, and the degree of FI was expressed by the “gray value” parameter with the help of professional image processing software.

Compared with the demographic data between the selected ASD patients and the control group, there was a statistically significant difference in BMD between the two groups. This is consistent with the results of previous studies [25]. In this study, the CT value of the cancellous area of the vertebral body was obtained to represent the patient’s BMD. Previous studies have confirmed that the CT value of vertebral cancellous bone is positively correlated with the actual patient’s BMD [26]; therefore, it is feasible to compare and analyze the CT value. The reason for this difference may be that patients with a lower BMD are more likely to have bone degeneration, destruction and spinal imbalance, and these patients have a higher risk of developing ASD than their peers after the first operation. This is consistent with previous perceptions that BMD is an important indicator that must be considered in patients undergoing spinal surgery at any stage.

**Fig. 3:**
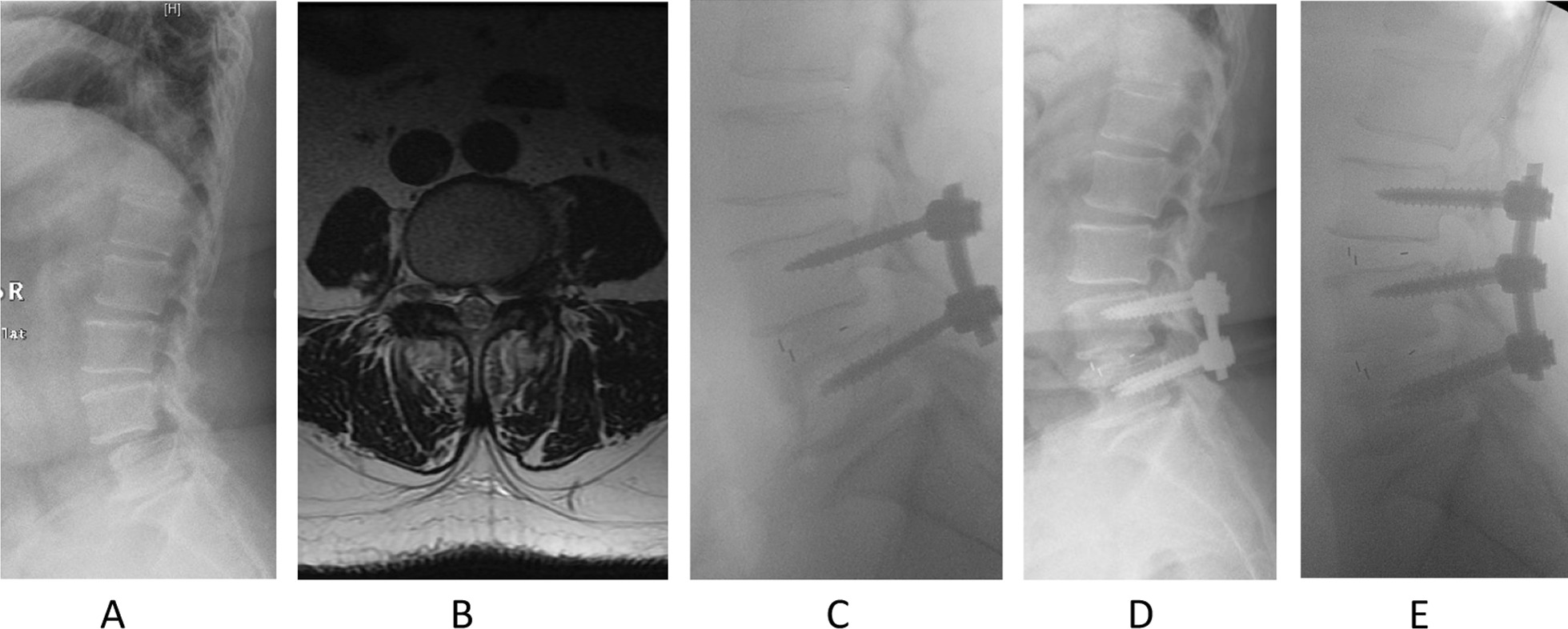
A 63-year-old female underwent L4–5 transforaminal lumbar interbody fusion surgery in 2016 for lumbar spondylolisthesis and lumbar spinal stenosis and underwent L3–4 revision surgery for adjacent segment disease 4 years later. **A** shows the lumbar lateral plain radiograph before the first surgery. **B** suggests significant fatty infiltration of the multifidus muscle at the L4–5 segment before the first surgery. **C** shows the lumbar lateral plain radiograph after the first surgery. **D** and **E** are pre- and postoperative radiographs of the revision surgery, respectively

We compared and analyzed the paraspinal muscle parameters of each intervertebral disk level (L1–S1) between the two groups. The results showed that there were significant differences in the rCSA and rFI of the MF muscle at the lower lumbar level between the two groups, but there was no significant difference in the quality and quantity of the ES muscle at each segmental level. This result confirms the important role of MF muscle in the development of ASD in patients, and the MF muscle at the lower lumbar level is more representative of the characteristics of the whole muscle. Then, we used univariate regression analysis to confirm that the smaller CSA of the MF muscle at L3–L4, the greater degree of FI at L4–L5 and L5–S1 and the lower BMD were all risk factors for ASD. It is worth noting that previous studies have shown a correlation between BMD and the degree of paraspinal muscle FI [27–29]. However, this correlation is not reflected in this study, considering that all subjects included in this study are patients with lumbar degenerative diseases, which is different from the normal people in previous studies. Figure 3 shows representative images of a woman who underwent revision surgery for ASD, which can be used as support for this conclusion.

Previous scholars have confirmed the effect of the number of segments in the first operation on the development of ASD [30–32]. We tried to divide all the patients into two groups according to the segment of the first operation and found that there were significant differences in the CSA and the degree of FI of the lower lumbar MF muscle between the two groups in the patients receiving single segment surgery for the first time. In the patients who received two-segment fusion for the first time, there was only a difference in the FI of the lower lumbar MF muscle between the two groups, but there was no difference in the CSA between the two groups. This may be because the longer fusion segment itself is a risk factor for the occurrence of ASD; on this basis, early changes in the paraspinal muscles can lead to the occurrence of ASD. Therefore, it can be considered that the FI of paraspinal muscles can reflect the changes in muscle characteristics more sensitively than the CSA. In actual clinical work in the future, it may be possible to directly determine the degree of FI of the lower lumbar MF muscle through intelligent image analysis software, which can be used as an evaluation index.

This study revealed the role of paraspinal muscles in the development of ASD. First, to prevent the occurrence of ASD, spinal surgeons should have a long-term vision, and the state of paraspinal muscles should also be taken into consideration when making surgical plans. Second, we should continue to emphasize the importance of low back muscle exercise and even set up a systematic exercise program for patients after the first lumbar fusion. Previous prospective studies on other spinal diseases have also shown that low back muscle exercise can effectively improve muscle quality [33–35].

This study also has some limitations. The sample size of this study was small, and there were only 33 cases in the ASD group. This is because there is a requirement for high-quality image data before the first operation. These images must be collected in this research center, hence limiting the number of sample size. In this study, all the segmental levels of each patient’s muscles were measured, compared and analyzed, which could have compensated, to a certain degree, for the small sample size. In addition, previous studies have shown that spinal-pelvic sagittal imbalance is also one of the risk factors for ASD [36, 37]. However, due to the overall lack of full-length films of the spine in our center, the measurement of pelvic parameters is limited.

## Conclusions

The CSA and FI of the lower lumbar MF muscle and BMD before the first operation were closely related to the occurrence of ASD after lumbar fusion. The CSA of the MF muscle at L3–L4, the FI of the MF muscle at L4–L5 and L5–S1 and BMD were important risk factors for the development of ASD. The number of fusion segments in the first operation had a certain impact on the above-mentioned conclusions, so the specific impact of the number of different fusion segments needs to be further studied.

## Data Availability

The datasets used and/or analyzed during the current study are available from the corresponding author on reasonable request.

## Abbreviations

ASD: Adjacent segment disease
CSA: Cross-sectional area
FI: Fat infiltration
MF: Multifidus
ES: Erector spinae
LL: Lumbar lordosis
SS: Sacral slope
BMD: Bone mineral density
BMI: Body mass index
rCSA: Relative cross-sectional area
rFI: Relative fat infiltration
